# Technical note: Providing proton fields down to the few‐MeV level at clinical pencil beam scanning facilities for radiobiological experiments

**DOI:** 10.1002/mp.15388

**Published:** 2021-12-22

**Authors:** Carina Behrends, Christian Bäumer, Nico Verbeek, Jens Ehlert, Rajendra Prasad, Jörg Wulff, Armin Lühr, Beate Timmermann

**Affiliations:** ^1^ West German Proton Therapy Centre Essen (WPE) 45147 Essen Germany; ^2^ Department of Physics TU Dortmund University 44227 Dortmund Germany; ^3^ West German Cancer Centre (WTZ) University Hospital Essen 45147 Essen Germany; ^4^ German Cancer Consortium (DKTK) 69120 Heidelberg Germany; ^5^ Faculty of Medicine University of Duisburg‐Essen 45147 Essen Germany; ^6^ Institute for Laser and Plasma Physics Heinrich‐Heine‐University Düsseldorf 40225 Düsseldorf Germany; ^7^ Department of Haematology, Oncology and Clinical Immunology, Heinrich‐Heine‐University Düsseldorf Medical Faculty 40225 Düsseldorf Germany; ^8^ Department of Particle Therapy University Hospital Essen 45147 Essen Germany; ^9^ Present address: Deutsches Elektronen‐Synchrotron DESY Hamburg Germany

**Keywords:** energy distributions, high LET, Monte Carlo, proton therapy, radiobiology, α‐particle range

## Abstract

**Purpose:**

The adequate performance of radiobiological experiments using clinical proton beams typically requires substantial preparations to provide the appropriate setup for specific experiments. Providing radiobiologically interesting low‐energy protons is a particular challenge, due to various physical effects that become more pronounced with larger absorber thickness and smaller proton energy. This work demonstrates the generation of decelerated low‐energy protons from a clinical proton beam.

**Methods:**

Monte Carlo simulations of proton energy spectra were performed for energy absorbers with varying thicknesses to reduce the energy of the clinical proton beam down to the few‐MeV level corresponding to μm‐ranges. In this way, a setup with an optimum thickness of the absorber with a maximum efficiency of the proton fluence for the provisioning of low‐energy protons is supposed to be found. For the specific applications of 2.5–3.3 MeV protons and α‐particle range equivalent protons, the relative depth dose was measured and simulated together with the dose‐averaged linear energy transfer (LETd) distribution.

**Results:**

The resulting energy spectra from Monte Carlo simulations indicate an optimal absorber thickness for providing low‐energy protons with maximum efficiency of proton fluence at an user‐requested energy range for experiments. For instance, providing energies lower than 5 MeV, an energy spectrum with a relative total efficiency of 38.6% to the initial spectrum was obtained with the optimal setup. The measurements of the depth dose, compared to the Monte Carlo simulations, showed that the dosimetry of low‐energy protons works and protons with high LETd down to the range of α‐particles can be produced.

**Conclusions:**

This work provides a method for generating all clinically and radiobiologically relevant energies – especially down to the few‐MeV level – at one clinical facility with pencil beam scanning. Thereby, it enables radiobiological experiments under environmentally uniform conditions.

## 1 INTRODUCTION

Radiobiological experiments are of great interest in radiotherapy research as they can provide information on microscopic processes and the biological effects of radiation. On the one hand, the radiobiological comparison of energy deposition and radiation‐induced late side effects between protons and photons is investigated.^1^, ^2^ On the other hand, there is research interest to compare high linear energy transfer (LET) protons with α−radiation,^3^, ^4^ carbon ions,^5^ or laser‐accelerated protons (LAP).^6^, ^7^, ^8^

Although clinical proton facilities can provide energies from about 60–230 MeV, in particular, the low‐energy protons are interesting for these radiobiological experiments, due to their high LET: At the distal end of the dose deposition (Bragg peak), the LET increases sharply. As the relative biological effectiveness is a function of LET, a usually clinically assumed constant value of 1.1 is an oversimplification and the resulting uncertain dose calculation is thought to be one of the possible reasons for normal tissue toxicities ^9^. To guide novel treatment planning and provide accurate models for variable relative biological effectiveness, other groups have employed cell survival studies to investigate proton high LET.^10^, ^11^, ^12^

To properly perform these studies, high LET proton fields must be provided. As the proton beam of Dahle et al.^10^ did not match the clinical beam quality and Chaudhary et al.^11^ used an eyetreatment beam line, there is still a need for achieving low‐energy proton fields in clinical pencil beam scanning (PBS) facilities. In the studies on biological effectiveness, Guan et al.^12^ and Patel et al.^13^ used energy‐attenuating components to produce low‐energy spectra with high LET from a clinical beam line. An in‐silico dosimetric characterization to study degraded proton energies for a small animal irradiation platform was presented by Gerlach et al.^14^

The procedure of energy‐degrading of clinical proton beams to a few MeV by placing an absorber in the beam path is, however, nontrivial, due to range straggling and decreasing fluence: Both effects become more pronounced with absorber thickness. In addition, the large number of scattering events results in a broad energy spectrum. These physical aspects affect the shape of the low‐energy spectra and suggest an optimization problem in the setup.

In this work, resulting energy spectra after various energy‐degrading setups and the evolution of the resulting energy distributions with absorber thickness were investigated using Monte Carlo (MC) simulations. This method addresses the generation of the highest fluence in association with an optimal absorber thickness, which is especially important in the energy range of a few MeV. Additional depth dose simulations were compared with dose measurements and LET studies were provided.

This work presents an approach for a low‐energy proton beam irradiation setup at a clinical PBS facility. Moreover, all proton energies of biological interest from proton ranges to treat patients down to the limit of traversing a mono‐layer cell culture were realized, all being available at one clinical facility to ensure stable environmental conditions. After applying the method in this work, a specific energy range can be filtered out from the resulting energy spectra using magnetic fields. Downstream of two magnets (one for energy selection and one for deflection in beam direction), a position‐dependent small energy range can be selected from the resulting low‐energy spectrum.^8^

The approach was applied exemplary for the comparison of clinically accelerated protons to LAP in the energy range of 2.5–3.3 MeV at the cell port center from the laser system ARCTURUS, University of Düsseldorf, Germany.^15^, ^16^ The acronym LAP is used below as a description for this specific energy range.

## 2 THEORETICAL ASPECTS

When protons interact with the absorber material, energy deposition, scattering, and also increased range straggling occur. The calculation of the resulting energy spectrum has already been mathematically analyzed earlier.^17^, ^18^ Here, it is sufficient to study the tendency of the increased range straggling with higher proton kinetic energy E, material thickness x, and the atomic number Z of the absorber material. According to Lewis^19^ and following Bohr's theory (1915),^20^ the total range straggling $\sigma_{R}^{2}$ defined as the variance of the total range distribution can be calculated as

(1) $$\sigma_{R}^{2}=4\pi nZe^{4}\int_{0}^{E_{0}}dE{\left(\frac{dE}{dx}\right)}^{-3}\frac{1-\beta^{2}/2}{1-\beta^{2}},$$

where n is the number of atoms per unit volume in the absorber, e is the proton charge, dE/dx is the stopping power, $E_{0}$ the initial proton energy, and $\beta^{2}=1-{\left(1+E/m_{p}c^{2}\right)}^{-2}$ with the proton mass $m_{p}$ and speed of light c.

Bichsel et al.^17^ and Tschalär et al.^18^ demonstrated that the resulting energy distribution is a function of the absorber thickness and the associated energy loss: For thin absorbers and energy loss up to 20% of the initial energy, the energy distribution agrees with Bohr's theory.^18^ With increased energy loss up to 80%, the spectra are described by the Landau distribution and their asymmetry increases.^21^ For energy losses greater than 80%, only a part of the initial particles contribute to the spectrum, because most of them are stopped in the absorber, which leads to a skewed distribution^18^, ^22^ (for illustration, see the energy distributions in Figure 1). Due to this change in the energy spectrum's shape and the decreasing fluence with increasing energy loss, an optimal absorber thickness is expected to achieve the highest fluence in the low‐energy range.

**FIGURE 1:**
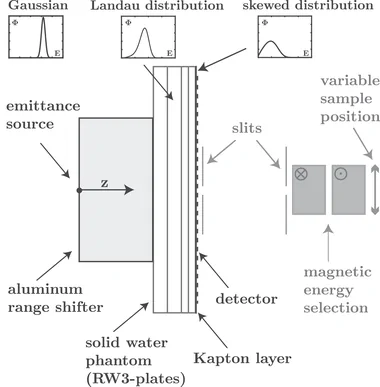
Schematic sketch of the experimental and MC simulation setup with different absorber thicknesses (black part). The aluminum range shifter has an extension of 38.993 mm and the solid water plates vary in thickness. During the measurements, additional Mylar foils (50 μm) were positioned downstream of the RW3‐plates, depending on the step size. The Kapton layer represents the scoring geometry of the simulation and the entrance of the extrapolation IC in the measurements, respectively. To illustrate the development of the energy spectra with absorber thickness, they are sketched above17,18: The source is a Gaussian energy distribution, and as it passes through absorber material, it evolves into the Landau distribution, which has a low‐energy tail. With a thick absorber, the direction of the skewness of the distribution changes and there are more low‐energy protons compared to a thin absorber. In addition to the setup used in this work, a possible energy selection system (gray part) is indicated with two slits and two magnets of opposite field directions, which results in a position‐dependent proton energy, based on.8,15 The variable position of the sample behind the magnets allows irradiation with a selected energy

## 3 MATERIALS AND METHODS

The clinical facility West German Proton Therapy Centre Essen (WPE) is equipped with an IBA ProteusPlus proton therapy system (IBA PT, Louvain‐la‐Neuve, Belgium) consisting of an isochronous cyclotron that accelerates protons to an energy of 226.7 MeV. With the energy‐selection system, the energies can be reduced down to 100 MeV. The investigations were performed for the horizontal beam line with the so‐called universal nozzle, operated in the PBS mode. Range shifter blocks can be mounted on the nozzle using the snout holder.

### 3.1 Experimental setup for the depth dose measurements

A range shifter block of aluminum (material composition: 94.8% aluminum, 4.5% magnesium and 0.7% manganese, thickness = 38.993 mm), solid water phantoms (RW3‐plates, type SP34 IBA Dosimetry, composition: 98% polystyrene + 2% TiO2) of nominal thicknesses between 1 and 10 mm, and 50 μm Mylar foils were used as absorber materials and to obtain fine steps in the measurements of the depth dose curve (DDC). To achieve high accuracy, the physical thickness of the aluminum block and each solid water plate was verified with a precision of 1 μm with the TESA‐HITE magna 400 IP40 (Tesa Technology).

An air‐filled extrapolation ionization chamber (IC) type 23391 (PTW, Freiburg, Germany) was used for depth dose measurements. The measured quantity was the released charge in the air volume, which is a measure for dose. This IC is well suited for the application of a few‐MeV protons because the entrance window, consisting of a stretched Kapton (polyimide) foil surrounded by aluminum, is only 25 μm thick. Thus, the relative dose of protons with very small residual ranges can still be detected. The IC was used with an electrode diameter of 3 cm, a fixed electrode gap of 2 mm, and a voltage of 300 V. The alignment was performed with a lateral accuracy of 0.5 mm using the clinical X‐ray system. For more information on the extrapolation IC, Kern et al.^23^ can be referred to.

The physical thicknesses of the absorber materials were converted into water equivalent thicknesses using the water equivalent ratio (WER). Table 1 gives an overview of the estimated WER of the different absorber materials used for the experimental water equivalent depth (WED). The energy‐dependent WER from RW3‐plates, Kapton and Mylar, was estimated by averaging of depth dose simulation and calculation. The WER of the aluminum alloy was determined by averaging experimental depth dose data and simulation. The measurement was performed by irradiating the unique multilayer ion chamber Giraffe (IBA PT, Louvain‐la‐Neuve, Belgium) with a single spot of 120 MeV and evaluating the pullback of $R_{80}$ (range at 80% dose) determined with a Bortfeld‐fit.^24^

**TABLE 1:** WER values of the different absorber materials used in the measurements for the water equivalent thickness

Material	Water equivalent ratio (WER)
Aluminum alloy	2.093
RW3‐plates	1.028
Kapton	1.309
Mylar	1.329

Figure 1 shows the experimental setup structure with the absorber materials and the detection plane indicating the position of the extrapolation IC. In addition, the figure suggests a possible energy selection system using a vacuum chamber following the method shown here (gray part): A slit aperture is located both at the entrance of the vacuum chamber and directly in front of the magnets. These are to limit and define the lateral extension of the proton beam entering the magnets.^8^, ^15^ However, the following results refer only to the setup shown in black with the scoring directly behind the absorber material components.

The initial proton energy was 120 MeV to measure the Bragg peak and normalize the data. To ensure that all protons emerge from the absorber with nearly the same energy, a scanned field of size 2×2 ${\mathrm{cm}}^{2}$ and spot spacing of 1 mm was designed: Due to the continuously increasing of the path length in the absorber with incoming deflection angle resulting from the scanning divergence, the outer protons were given a correspondingly higher energy.

### 3.2 TOPAS Monte Carlo simulations

In the Geant4 TOol of PArticle Simulation (TOPAS),^25^ the energy spectra as well as a DDC and dose‐averaged LET (LETd) were simulated. The Fermi–Eyges parameter, mean energy and energy spread, of the emittance source were estimated matching the properties of the proton beam of the WPE.^24^, ^26^, ^27^, ^28^ The simulation source was placed directly at the upstream surface of the first absorber (see Figure 1). For each simulation, $10^{9}$ primary particles were sampled.

The same material composition of the aluminum alloy was used as for the measurements, with a density of 2.66 g/$cm^{3}$.^29^ The composition of RW3 as specified above resulted in a density of 1.045 g/$cm^{3}$. Based on ICRU Report 90,^30^ a mean excitation energy of 166.0 eV for aluminum and 78 eV for water was chosen. For the calculation of the electronic stopping powers, TOPAS uses ICRU Report 49^31^ and ICRU Report 90^30^ by default, where the latter applies to water.

The production cut‐off for all particles was set to the range of protons with an energy of 1 MeV in water, which corresponds to $2.458\times 10^{-3}$ cm range in water based on the continuous slowing down approximation of the National Institute of Standards and Technology (NIST).^32^ For the maximum step size, the default value of 1 mm was maintained. In analogy to Jarlskog et al.,^33^ *g4em‐standard opt4, g4hphy QGSP BIC HP, g4decay, g4ion‐binarycascade, g4h‐elastic HP*, and *g4stopping* were used as physics packages. No variance‐reduction strategies were employed.

For the energy spectra simulations, a central spot was used. Aluminum, RW3‐plates and a 12.5 μm Kapton layer were investigated as absorber materials. The variation of the absorber thickness was implemented only via different RW3‐plate combinations. The additional use of the Kapton layer resulted from the possible application with the energy selection system (window of vacuum chamber, see gray part in Figure 1). The proton fluence including the standard deviation was tallied in a cylindrical volume with a radius of 10 cm placed directly behind the absorber material (see Figure 1).

The same 2×2 ${\mathrm{cm}}^{2}$ scanned field from the measurements was used for the simulation of the DDC and the LETd. Only the aluminum block and a subsequent cylindrical volume of water were used, which served as absorber material and detector in WED. The latter had the electrode radius of the extrapolation IC and tallied the dose to medium and the LETd as well as the standard deviations in 0.05 mm bins in depth. Based on Granville et al.^34^ and Cortés‐Giraldo et al.,^35^ the dE/dx‐computation technique was employed using the electronic stopping power for the prestep proton energy to calculate the LETd. The secondary electron production threshold, which has a minor influence on the results in the used dE/dx‐calculation technique, was set to 1 mm.^34^, ^35^

### 3.3 Uncertainty analysis

Experimental and simulation uncertainties result in type A and type B uncertainties.^36^ Thereof, only the statistical variations of the measured and the simulated values were treated as type A uncertainties. The other contributions to uncertainty analysis were considered as type B uncertainties with a rectangular probability distribution from which the standard deviation was calculated with the coverage factor of $\frac{1}{\sqrt{3}}$.

Regarding the experimental uncertainties, the maximum statistical uncertainty of the scored charge with the extrapolation IC yielded 0.9%. The other experimental uncertainties concerned the determination of the WED: Facility‐dependent uncertainties resulting in energy fluctuations of 0.05 MeV^29^ corresponded to 0.08 mm range in water for 120 MeV protons (NIST).^32^ There was also an uncertainty in the physical thickness of the absorber materials used, which affected the resulting WED. The maximum variation in water equivalent thickness of the different RW3‐plates and Mylar combinations used in the measurements was ≤0.1 mm and of the aluminum block 0.005 mm. Additional air scattering due to unavoidable air spaces between the absorber and the IC was neglected. The total experimental error in WED resulted from the quadratic addition of all components.

In the simulations, the maximum standard statistical uncertainty above the fluence value of 10% with regard to the maximum of the respective energy spectrum was 1.1%. For the simulations of the DDC, the average standard statistical uncertainty of the dose was less than 0.1% in all cases. Regarding the uncertainties in depth, the bin size of 0.1 MeV resulting in 0.002 mm range in water was given for the energy spectra simulations and the bin size of 0.05 mm was given for the depth dose and the LETd simulations.

## 4 RESULTS

Figure 2 gives an overview of the relative efficiency as a function of energy and WED of 120 MeV protons after traversing the stack of absorber material combinations. Relative efficiency is defined as the number of protons resulting from the scored fluence in relation to the initial number of protons. From now on, it is mainly referred to the efficiency. In Figure 2(a), the evolution of the shape of the spectra with increasing absorber thickness can be seen. As soon as some protons stop in the absorber, the Landau tail disappears and the spectrum becomes skewed. For the energy range of ≤5 MeV (including the LAP range discussed here as a special application), an absorber layer of water equivalent 107.4 mm shows the highest efficiency (magenta line): The inset in Figure 2(a) indicates the magnified section of the spectra in the energy range per nucleon up to 4 MeV/u. For use in radiobiology, the proton energy limit of 0.9 MeV required to irradiate 20 μ m thick cells is sketched. Furthermore, the marked 2.5–3.3 MeV proton energy range is close to the energy per nucleon of α‐particles coming from an americium source (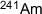).^37^ Thus, protons with the range of α‐particles can be produced.

**FIGURE 2:**
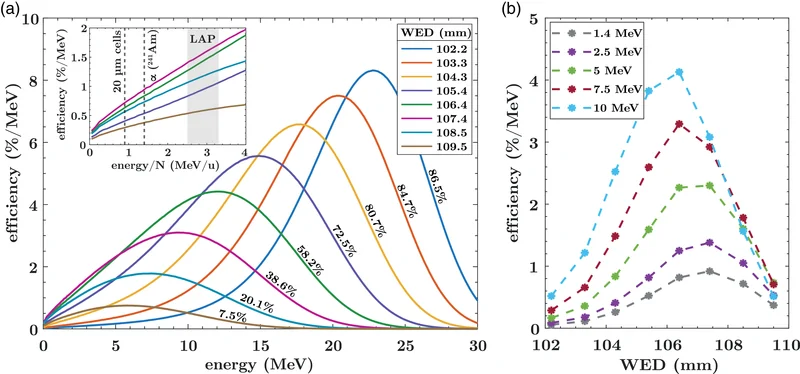
Simulated proton energy spectra after traversing different water equivalent absorber thicknesses with an initial energy of 120 MeV are presented in (a). As a measure of fluence, the efficiency relative to the initial particles as a function of energy is given on the ordinate. For all simulations, the 38.993 mm thick aluminum block was used. Different RW3‐plate phantoms were placed in addition to reach the presented WED. The cumulative WED of the absorber material is given in the legend (compare Figure 1). The numbers at the line plots indicate the total efficiency of the energy spectra to the initial spectrum. In the inset only, the energies per nucleon (N) up to 4 MeV/u are shown to demonstrate the efficiency in the low‐energy range. For the LAP, for example, the highest efficiency is achieved with an absorber thickness of 107.4 mm (magenta line). Besides, additional information concerning the energy range are given: The proton energy of 0.9 MeV is marked as the natural energy limit for the irradiation of 20 μm thick cells. The LAP's energy range is close to the energy per nucleon of α‐particles of about 1.4 MeV/u produced by an americium source.^37^ In (b), the relative efficiency as a function of energy in relation to the respective WED is shown for selected energies. Depending on the energy, an efficiency optimum results. The dashed lines are intended to guide the eye

Figure 2(b) presents the efficiency as a function of energy for different absorber thicknesses and selected energies. For each energy, there is an optimal setup where the maximum efficiency is achieved: For example, at the energy of about 2.5 MeV targeted energy for experiments, the maximum efficiency is at a WED of 107.4 mm. At this WED, an energy spectrum is obtained, which has a total efficiency of 38.6% compared to the initial spectrum (as seen in Figure 2a). With increasing user‐requested energy, the efficiency optimum shifts to thinner absorber thicknesses (see Figure 2b).

The depth dose and LETd simulation results as well as the measurement results are presented in Figure 3. The normalized measured values of the Bragg peak region fit with the error bars within the peak region of the simulation data and deviate only slightly (by about 0.1 mm in depth) in the distal edge. The maximum deviation in dose is 4% on the distal edge.

**FIGURE 3:**
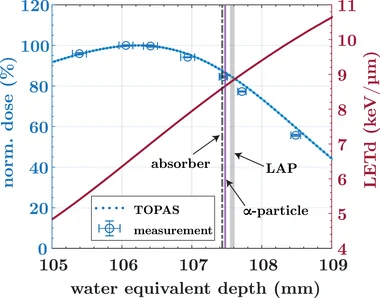
Simulated and measured depth dose together with the corresponding dose‐averaged LET (LETd) of initial 120 MeV protons. The simulated DDC and the measurement results belong to the left ordinate indicating the normalized dose to the maximum (blue). In the calculated DDC, the points show the values of the simulation linearly interpolated by the light blue line. The red curve displays the simulated LETd of the proton beam using the electronic stopping power for the prestep proton energy and belongs to the right ordinate, respectively. Furthermore, the dashed vertical line shows the position of the downstream end of the absorber material of the optimal setup (107.4 mm WED). The adjacent violet line indicates the residual range behind the estimated setup of protons with an energy per nucleon of 1.4 MeV range‐equivalent of α‐particles and the gray shaded area illustrates the ranges from the LAP, accordingly 2.5–3.3 MeV

As expected, the simulated LETd increases sharply with depth at the distal edge from the Bragg peak, reaching values above 10 keV/μm at the end of the DDC.

The dashed vertical line indicates the downstream end of the absorber material with 107.4 mm WED, which corresponds to the optimal setup for comparison with LAP from the analysis of the efficiency in Figure 2. The residual proton range in water after leaving the 107.4 mm absorber results in 42.0 μm (violet line) and an interval from 109.9 to 176.5 μm (gray area) for the proton energies of 1.4 MeV and of 2.5–3.3 MeV, respectively. This region of low‐energy range is just upstream the range $R_{80}$ (the range at which the fluence has fallen to approximately half) and thus in the distal fall‐off of the Bragg peak. Figure 3 also indicates that the LETd in the range of LAP equivalent clinical protons is about 9 keV/μm.

## 5 DISCUSSION

This study demonstrates the existence of an optimal setup with energy‐attenuating components for an efficient delivery of low‐energy proton spectra at a clinical facility. This was particularly illustrated for protons with energies lower than 5 MeV with initial 120 MeV. In addition, comparison of the measured and simulated depth dose showed that the measurements and simulations agree within 0.1 mm in depth and 4% in dose, respectively.

Compared to Guan et al.^12^ and Patel et al.,^13^ who also approached high LET with different absorber thicknesses, this work specifically considers the physical effects and experimental problems arising with very thick absorbing components in the beam path.

The shapes of the simulated energy distributions at different absorber thicknesses correspond to the work of Bichsel et al.^17^ and to Tschalär's theory.^18^ The increasingly asymmetric distributions produce an efficiency optimum for the application in the few‐MeV energy range. To interpret the existence of the efficiency maximum in the low‐energy range, range straggling has to be considered on the one hand, and the number of remaining protons on the other hand. The width of the energy spectrum resulting from range straggling increases with absorber thickness (compare Equation (1)). An increasing absorber thickness also results in an increasing number of protons giving up their entire energy in the material and thus no longer contribute to the spectrum, which means that the distribution becomes narrower again. As the optimal water equivalent absorber thickness for the considered setup is close to the range of the initial proton energy in water, the contributing particle number decreases sharply with increasing absorber thickness. This interplay of decreasing particle number and increasing range straggling leads to the efficiency maximum in the low‐energy range.

The change in the shape of the spectrum due to the more linear low‐energy slope can be explained by electronic ionizations increasing with low kinetic proton energy (see LETd in Figure 3). In other words, the increased LETd at lower proton energies leads to more low‐energy protons. Nuclear reactions will not make a decisive contribution, because the effective cross sections for nonelastic nuclear reactions for proton energies below 10 MeV are negligible.^38^

To improve the beam transmission for low‐energy protons, the absorber material should have an atomic number Z as low as possible, because both range straggling (see Equation (1)) and multiple Coulomb scattering scale with Z. It is also advisable to apply the outer proton spots with higher energy in an extended field according to their longer path length through the absorber. In this case, the off‐axis protons were given an energy too high for the absorber and the energies in the field went up to 120.7 MeV, but this was taken into account in the associated simulation. In addition, the absorber material should be practicable in application and in production. For the latter, especially the temperature dependence, but also the stability is of importance. In this case, the aluminum alloy was chosen to provide a good compromise between the requirements and manufacturing conditions. Even better conditions are offered by graphite and beryllium with beryllium showing a 40% higher beam transmission compared to carbon as a degrader material.^39^ As graphite is brittle and beryllium is biologically harmful, an outer layer of, for example, aluminum would be useful when using it as a range shifter.

This work compares simulations and measurements under extreme scattering and stopping conditions. The agreement between the two methods validates the accurate modeling of energy loss and range straggling of the TOPAS MC code.

The simulated LETd of 9 keV/μm with initial clinical energy is not very high in terms of radiobiological effects compared to other initial proton energies: In the work of Dahle et al.,^10^ an LETd of about 30 keV/μm in the $R_{80}$ region could be produced with initial 15.5 MeV and 15 keV/μm at clinical initial 80 MeV, respectively. A comparison with α‐particles shows about the same range as protons at the same energy per nucleon, but the stopping power is still about four times as high and so is the LETd (NIST).^32^ Hence, it is reasonable to choose an initial energy as small as possible to achieve a higher LETd.

Like the energy spectra shown here are characterized by mean proton energies, the LETd values are also obtained from LET spectra. Changes in such LET spectra were examined in more detail in Dahle et al.^10^ In principle, these become broader with increasing depth and initial energy. Nevertheless, differently shaped LET spectra can yield the same LETd value. Therefore, various initial energies and LET spectra can lead to different biological effects although yielding the same LETd value.^10^, ^11^ This aspect has to be considered in radiobiological experiments.

With regard to reproducibility, the measurement data were recorded consistently over a period of 1 month. If necessary, fluctuations of the energy or the range of the clinical protons from the cyclotron must be taken into account accordingly for this type of experiment.

When applying this method to other facilities with other types of proton accelerators, there may be differences in initial energy or energy spread: As already pointed out, it is generally advantageous to use an accelerator with a low initial energy. Compared to a cyclotron, the energy spread will be smaller for a proton synchrotron, because the latter has no degrader. The synchrotron at the Heidelberg Ion Beam Therapy Center (HIT), for example, has a momentum spread of 0.3% at low energies,^40^ which is roughly a factor of 3 smaller than that of the cyclotron used. Accordingly, the use of a synchrotron with lower energy spread will lead to narrower energy spectra and thus higher proton fluences per energy. Nevertheless, no fundamental change in the approach of this work is expected if the accelerator parameters of another facility vary. The existence of an optimal setup for maximum efficiency of low‐energy protons is still reasonable.

Dosimetry of low‐energy protons presents a particular challenge, although dosimetry was not the primary goal of this work. Clinical detectors typically have a too thick entrance window to measure these low proton energies employed here. Nevertheless, there are alternatives to the appropriate extrapolation IC, such as radiochromic film dosimetry,^41^ which, however, has large uncertainties,^23^ as well as calibrated photostimulated image plates or solid state detectors CR39.^42^

A special application of this work was to find the ideal setup at a clinical proton accelerator for low‐energy experiments to perform cell irradiations comparable to the specifically presented case with LAP.^15^ As the setup with the mentioned energy selection system is much more complex than assumed in this work, a further simulation was required. Additional scattering effects due to the slit apertures dominating the resulting energy spectrum at the optimal setup should be excluded. The simulation showed that the shape of the energy spectrum itself is not affected, but the efficiency is considerably reduced: Behind the second slit aperture, only 0.01% of the particles without an aperture were detected. This low yield for an applicable setup underlines the pertinence of the presented method in this work to find the optimal efficiency behind the absorber material components.

Further applications of this work may be radiobiological studies comparing the low‐energy protons and LAP or α‐particles due to their similar range, as well as experiments with high LET protons in contrast to carbon ions or corresponding relative biological effectiveness investigations. Beyond that, this setup could be used to study the response functions of different detectors at the end of the beam range.

Overall, this work provides a meaningful step for degrading clinical proton beams, but does not solve all the practical issues associated with this method. Future work should explore the following challenges: One challenge is providing an adequate dose rate for cell experiments due to the high proton loss through the absorber and potentially used apertures. Fundamental issues like an accurate target arrangement and the already mentioned appropriate beam dosimetry of low‐energy protons need to be considered. In addition, it is known from Trinkl et al.^43^ that more secondary neutrons are produced when using a range shifter. Therefore, the downstream neutron contamination by the thick absorber must be additionally investigated in cellular dosimetry.

## 6 CONCLUSIONS

This work presents an approach for generating low‐energy protons at a clinical facility. It has been shown that there is an optimum absorber thickness to reduce the energy of the protons accordingly and achieve high efficiency. The identified WED for the application of protons ≤5 MeV corresponds to a position at the distal flank of the Bragg peak where the LET of the protons is correspondingly high. At this point, the decreasing number of protons and increasing range dispersion result in an efficiency maximum in the relevant energy range. Nevertheless, there are further practical challenges to be investigated for degrading clinical proton beams for radiobiological experiments.

## CONFLICT OF INTEREST

The authors declare no conflict of interest.

## Data Availability

The data that support the findings of this study are available from the corresponding author upon reasonable request.
